# Should a prolonged duration of dual anti-platelet therapy be recommended to patients with diabetes mellitus following percutaneous coronary intervention? A systematic review and meta-analysis of 15 studies

**DOI:** 10.1186/s12872-016-0343-y

**Published:** 2016-08-30

**Authors:** Pravesh Kumar Bundhun, Chandra Mouli Yanamala, Feng Huang

**Affiliations:** 1 Institute of Cardiovascular Diseases, the First Affiliated Hospital of Guangxi Medical University, Nanning, Guangxi 530021 People’s Republic of China; 2 Department of Internal Medicine, EALING Hospital, University of Buckingham, Uxbridge road, Southall, UB1 3HW London, UK

**Keywords:** Dual antiplatelet therapy, Percutaneous coronary intervention, Diabetes mellitus, Drug eluting stents, Clopidogrel, Stent thrombosis, Bleeding events

## Abstract

**Background:**

This study aimed to compare the adverse clinical outcomes associated with a short and a prolonged duration of Dual Anti-Platelet Therapy (DAPT) in patients with Diabetes Mellitus (DM) after undergoing Percutaneous Coronary Intervention (PCI).

**Methods:**

Medline/PubMed, EMBASE and the Cochrane library were searched for studies comparing the short and prolonged DAPT use in patients with DM. Adverse outcomes were considered as the clinical endpoints in this analysis. Odds Ratios (OR) with 95 % Confidence Intervals (CI) were used to express the pooled effect on discontinuous variables and the pooled analyses were performed with RevMan 5.3.

**Results:**

Fifteen studies with a total number of 25,742 patients with DM were included in this current analysis which showed no significant differences in primary endpoints, net clinical outcomes, myocardial infarction and stroke with OR: 1.03, 95 % CI: 0.65–1.64; *P* = 0.90, OR: 0.96, 95 % CI: 0.69–1.34; *P* = 0.81, OR: 0.85, 95 % CI: 0.70–1.04; *P* = 0.12 and OR: 0.94, 95 % CI: 0.65–1.36; *P* = 0.75 respectively. Revascularization was also similar between these 2 groups of patients with DM. However, even if mortality favored prolonged DAPT use, with OR: 0.87, 95 % CI: 0.76–1.00; *P* = 0.05, the result only approached significance. Also, stent thrombosis insignificantly favored a prolonged DAPT duration with OR: 0.56, 95 % CI: 0.27–1.17; *P* = 0.12. Thrombolysis In Myocardial Infarction (TIMI) defined major and minor bleeding were not significantly different in these diabetic patients with OR: 0.91, 95 % CI: 0.60–1.37; *P* = 0.65 and OR: 1.08, 95 % CI: 0.62–1.91; *P* = 0.78 respectively. However, bleeding defined by the Bleeding Academic Research Consortium (BARC) classification was significantly higher with a prolonged DAPT use in these diabetic patients with OR: 1.92, 95 % CI: 1.58–2.34; *P* < 0.00001.

**Conclusion:**

Following PCI, a prolonged DAPT use was associated with similar adverse clinical outcomes but with a significantly increased BARC defined bleeding compared to a short term DAPT use in these patients with DM. However, even if mortality and stent thrombosis favored a prolonged DAPT use, these outcomes only either reached statistical significance or were insignificant respectively, showing that a clear decision about recommending a prolonged duration of DAPT to patients with DM might not be possible at this moment, warranting further research in this particular subgroup.

## Background

According to guidelines, Dual Anti-Platelet Therapy (DAPT) with aspirin and P2Y12 inhibitors, mainly clopidogrel, is recommended for at least one year following Percutaneous Coronary Intervention (PCI) with Drug Eluting Stents (DES) [1]. However, even in this new era, several Randomized Controlled Trials (RCTs) still could not predict the exact duration of DAPT use and suggested that this issue might possibly be solved only using a larger number of randomized patients following PCI with DES [2]. To be more clear, in a previously published study comparing 6 months with 12 months DAPT use, the authors stated that larger trials would be able to completely solve this issue [3]. Even if several meta-analyses comparing the short and prolonged DAPT use in the general population following PCI showed a longer duration of DAPT to be associated with favorable clinical outcomes [4], other meta-analyses showed no benefits of a prolonged DAPT duration [5] giving rise to controversies. However, whether these results also apply to the subgroup of patients with Diabetes Mellitus (DM) have seldom been studied. Therefore, this study aimed to compare the adverse clinical outcomes associated with a short and prolonged duration of DAPT use in patients with DM following PCI.

## Methods

### Data sources and search strategy

Medline/PubMed, EMBASE and the Cochrane library were searched for studies comparing the short and prolonged use of DAPT in patients with Acute Coronary Syndrome (ACS) following PCI by typing the words ‘dual anti-platelet therapy, diabetes mellitus and percutaneous coronary intervention’. Another search was performed using the phrase ‘prolonged clopidogrel use, diabetes mellitus and percutaneous coronary intervention’. To widen this search, the abbreviations ‘DAPT, DM, and PCI’ as well as the term ‘coronary angioplasty’ were also used. In addition, reference lists of suitable studies were also reviewed for relevant articles. Only articles published in English were considered in this search process.

### Inclusion and exclusion criteria

Studies were included if:

- They were randomized trials or observational studies.
- They compared short and prolonged DAPT use and included patients with DM.
- They reported adverse outcomes as their clinical endpoints.
- They were published in English.

Studies were excluded if:

- They were meta-analyses, case studies or editorials.
- They did not involve patients with DM.
- They did not report adverse outcomes as their clinical endpoints.
- They did not compare short with prolonged DAPT use, but instead, compared aspirin monotherapy with DAPT following PCI.
- They were duplicates or involved the same trial.

### Definitions, outcomes and follow up

DM was defined as a state of high blood sugar levels observed at least on two separate occasions, with a fasting blood glucose test or an oral glucose tolerance test, with or without symptoms (asymptomatic) such as polydipsia (frequent thirst), polyuria (frequent urination) and weight loss.

Adverse clinical outcomes which were analyzed in this study included:

- Primary endpoint which was a composite endpoint of all-cause death, Myocardial Infarction (MI), stroke, revascularization and stent thrombosis.
- MI (any type or any classification of MI) was relevant including the universal definition [6].
- Target Lesion Revascularization (TLR).
- Target Vessel Revascularization (TVR).
- All-cause death (cardiac and non-cardiac).
- Stroke.
- Net Adverse Clinical and Cerebral Events (NACCE) were defined as a composite of all-cause death, all MI, stroke or major bleeding.
- Stent thrombosis which was defined by the Academic Research Consortium (ARC) [7].
- Bleeding:
- All/Any bleeding.
- Major and Minor bleeding defined by Thrombolysis in Myocardial Infarction (TIMI) [8].
- Bleeding defined according to the Bleeding Academic Research Consortium (BARC) [9] which was further divided into BARC type 2, BARC type 3 and BARC type 5.

The adverse clinical outcomes reported have been listed in Table 1.

**Table 1:** Reported outcomes

Studies	Reported outcomes	Follow up period	Bias grade
Brar2008	Death, MI	9.7 months	-
I-LOVE IT 2	NACCE, death, MI, stroke, TVR, TLR, ST, all bleeding, major bleeding	12 and 18 months	B
ISAR-SAFE	Primary endpoints, death, MI, ST, stroke, TIMI major and minor bleeding, BARC bleeding	9 months	B
Tarantini2016	Death, MI, composite endpoints, stent thrombosis, BARC type 3 or 5 bleeding, stroke, revascularization	1 year	-
ARCTIC	Primary endpoints, Death, MI, ST, stroke, revascularization	17 months	B
OPTIMIZE	NACCE, death, MI, stroke, ST, major bleeding, TLR, TVR, any bleeding	1 year	B
RESET	Primary endpoints, death, MI, TVR, ST, major and minor bleeding, stroke	1 year	B
EXCELLENT	Death, MI, stroke, TVR, TLR, ST, any bleeding, TIMI major bleeding	1 year	B
PEGASUS	Death, MI, stroke, TIMI major and minor bleeding	3 years	B
DAPT	ST, MACCEs, death, stroke, MI, BARC type 2,3 or 5	12 to 30 months	B
Sardella2011	Death, MI, stroke, TIMI minor bleeding, revascularization	2 years	-
PRODIGY	Death, MI, stroke, ST, TLR, TVR, TIMI major and minor bleeding, BARC bleeding	2 years	B
Thukkani2015	Death, MI, Stroke	4 years	-
ENDEAVOR	Death, MI, ST (definite and probable), stroke, major bleeding	2 years	B
ITALIC	Primary endpoints, minor bleeding, minimal bleeding, death, MI, stroke, TVR, ST, major bleeding	1 year	B

*Abbreviations*: *MI* myocardial infarction, *ST* stent thrombosis, *TVR* target vessel revascularization, *TLR* target lesion revascularization, *NACCE* net adverse clinical and cerebral events, *BARC* bleeding academic research consortium, *TIMI* thrombolysis in myocardial infarction

### Short and prolonged duration of DAPT

Short and prolonged duration of DAPT use were based on the following criteria:

If the short term DAPT duration period was 3 months, its corresponding prolonged duration period should be more than 3 months (6, 12, 24, or more).

If the short term duration of DAPT was 6 months, its corresponding prolonged duration period should be more than 6 months (12, 18, 24, or more).

If the short term duration of DAPT use was 12 months, its corresponding prolonged duration period should be more than 12 months.

Therefore, a prolonged duration of DAPT was defined as the use of DAPT during a period of time longer than the actual short term duration corresponding to that particular trial. Different trials had different short and prolonged duration of DAPT use. Table 2 further illustrated the short and prolonged duration periods of DAPT use in each of the studies included in this meta-analysis.

**Table 2:** General features of the studies included

Features	No of DM patients in the short term group (*n*)	No of DM patients in the long term group (*n*)	Type of study	Enrollment period	Duration of DAPT use (months)
Brar2008	378	371	OB	2002–2004	<9 vs > 9
I-LOVE IT 2	211	203	RCT	2012–2015	6 vs 12
ISAR-SAFE	495	484	RCT	2008–2014	6 vs 12
Tarantini2016	206	223	RCT	2009–2014	6 vs > 12
ARCTIC	222	198	RCT	2009–2011	<12 vs >12
OPTIMIZE	554	549	RCT	2010–2015	3 vs 12
RESET	316	305	RCT	2009–2010	3 vs 12
EXCELLENT	272	278	RCT	2008–2009	6 vs 12
PEGASUS	1950	1574	RCT	2010–2013	<12 vs > 12
DAPT	1481	1556	RCT	2009–2011	12 vs 30
Sardella2011	133	139	OB	2005–2006	12 vs > 12
PRODIGY	35	36	RCT	2006–2012	6 vs 24
Thukkani2015	6568	5949	OB	2002–2006	12 vs > 12
ENDEAVOR	198	183	RCT	2005–2011	12 vs > 24
ITALIC	331	344	RCT	2011–2015	6 vs 24
Total no of patients (n)	13,350	12,392			

*Abbreviations*: *DM* diabetes mellitus, *DAPT* dual antiplatelet therapy, *RCT* randomized controlled trials, *OS* observational studies

### Data extraction and review

Two authors (PKB and CMY) independently assessed the studies involved and reviewed the methodological quality of each eligible trial. Information regarding the study/trial names, time period of patients’ enrollment, adverse clinical outcomes reported, the follow up periods, data concerning the total number of patients with DM classified into the short and prolonged DAPT groups respectively, the total number of clinical events reported in each subgroup, as well as information concerning the baseline features of the patients were carefully extracted and cross checked. During the data extraction process, any disagreement which occurred between these two authors was carefully discussed, and if they could not reach a consensus, the disagreement was solved and a final decision was made by the third author (FH). The bias risk among the trials (low risk, moderate risk and high risk) was assessed with the components recommended by the Cochrane Collaboration [10]. The six components of the bias risk were as follow:

A. Sequence generation
B. Allocation sequence concealment
C. Blinding of participants and personnel
D. Blinding of outcome assessment
E. Incomplete outcome data
F. Selective outcome reporting and other potential bias

The trials included in this study were analyzed according to these six components and a bias grade was given accordingly after a careful assessment. A grade ranging from A to E was considered whereby grade A was allocated if an extremely low risk of bias was reported, while a grade E was allocated if a very high risk of bias was observed. Note that these bias grades were just an approximation according to what the authors were able to assess. The bias risk grades allocated to each trial were provided in Table 1. Note that observational studies were ignored during this assessment.

### Methodological and statistical analysis

Recommendations of the PRISMA (Preferred Reporting Items for Systematic Reviews and Meta-Analyses) statement were followed in this study [11]. Heterogeneity among the subgroups was assessed using the Cochrane Q-statistic test whereby a P value less than 0 · 05 was considered statistically significant whereas *P* value ≥ 0.05 was considered statistically insignificant. I^2^-statistic test which also assessed heterogeneity, whereby an I^2^ with a low percentage (<25 %) represented a low heterogeneity, an I^2^ with a percentage between 25 and 50 % represented a moderate heterogeneity and an I^2^ with a high percentage above 50 % denoted an increasing heterogeneity. If I^2^ was less than 50 %, a fixed effect model was used during this subgroup analysis. However, if I^2^ was more than 50 %, a random effect model was used. Publication bias was visually estimated by assessing funnel plots. Odds Ratios (OR) with 95 % Confidence Intervals (CIs) were calculated for categorical variables and the pooled analyses were performed with RevMan 5.3 software. Ethical committee or medical institutional board approval was not required since this is a systematic review and meta-analysis of several studies.

## Results

### Search result

**Fig. 1:**
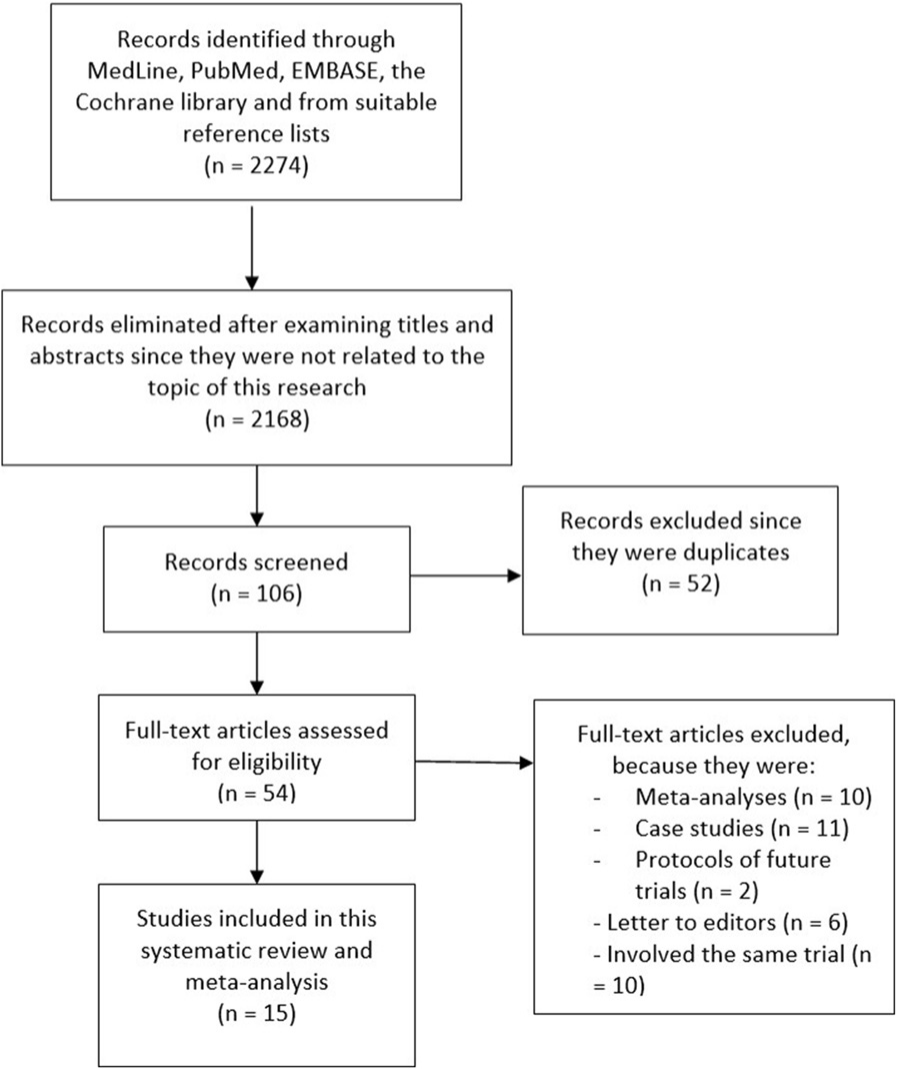
Flow diagram representing the study selection

Two thousand two hundred seventy four articles were obtained from PubMed/Medline, EMBASE, the Cochrane Library and from suitable reference lists. After a careful selection and assessment of titles and abstracts, 2168 articles were eliminated since they were not related to the topic of this research. Among the 106 remaining articles, 52 articles were further eliminated since they were duplicates. Fifty-four full-text articles were assessed for eligibility. Ten studies were further eliminated since they were meta-analyses, 11 studies were case studies, 2 studies were protocol of future ongoing trials, 6 articles were letter to editors, and 10 articles were associated with the same trial. Finally, 15 studies (Brar2008 [12], I-LOVE IT 2 [13], ISAR-SAFE [14], Tarantini2016 [15], ARCTIC [16], OPTIMIZE [17], RESET [18], EXCELLENT [3], PEGASUS [19], DAPT [20], Sardella2011 [21], PRODIGY [22], Thukkani2015 [23], ENDEAVOR [24], ITALIC [25]) that satisfied all the inclusion and exclusion criteria of this current analysis, were included. The flow diagram representing the study selection has been illustrated in Fig. 1.

Study Tarantini2016 [15] was a sub-study of the SECURITY trial [26] including patients only with DM and trials DES LATE [27] and REAL-LATE ZEST-LATE [28] were excluded because they compared aspirin monotherapy versus DAPT, instead of prolonged DAPT use versus short term DAPT use.

### General features of the studies included

A total number of 25,742 patients with DM (13,350 patients assigned to short term DAPT group whereas 12,392 patients assigned to prolonged DAPT group) were included. Patients were enrolled from the year 2002 to the year 2015. The general features of the studies included have been listed in Table 2.

### Baseline features of the studies included

Table 3 summarized the baseline characteristics of the patients included in this meta-analysis.

**Table 3:** Baseline features of the studies included

Studies	Mean age	Males (%)	HT (%)	Ds (%)	Cs (%)
	S/L	S/L	S/L	S/L	S/L
Brar2008	62.9/62.9	70.0/70.0	-	-	-
I-LOVE IT 2	60.4/60.0	67.2/68.7	61.0/64.8	25.3/23.4	24.2/24.9
ISAR-SAFE	67.2/67.2	80.7/80.5	90.1/91.5	87.5/87.4	24.9/25.7
Tarantini2016	65.5/66.7	71.8/74.0	82.5/80.3	69.4/70.9	18.9/20.2
ARCTIC	64.0/64.0	81.0/80.0	62.0/59.0	68.0/67.0	24.0/23.0
OPTIMIZE	61.3/61.9	63.5/63.1	86.4/88.2	63.2/63.7	18.6/17.3
RESET	62.4/62.4	64.4/62.9	62.3/61.4	57.7/59.9	25.2/22.8
EXCELLENT	63.0/62.4	65.1/63.9	72.7/73.8	75.2/76.3	27.4/25.8
PEGASUS	65.0/66.0	77.0/77.0	76.0/76.0	76.0/77.0	16.0/17.0
DAPT	61.6/61.8	74.0/75.3	74.0/75.8	-	24.7/24.6
Sardella2011	61.9/61.2	78.2/81.3	76.7/74.8	57.1/64.0	48.1/62.6
PRODIGY	70.0/68.0	75.0/78.0	71.0/72.0	62.0/66.0	12.0/15.0
Thukkani2015	64.3/64.3	98.5/98.5	97.1/97.8	-	34.8/33.3
ENDEAVOR	62.4/63.6	69.6/69.4	73.6/79.5	80.5/81.4	52.3/56.2
ITALIC	61.7/61.5	80.8/79.2	65.2/64.7	67.1/67.1	50.9/52.7

*Abbreviations*: *S* short term DAPT use, *L* prolonged DAPT use, *HT* hypertension, *Ds* dyslipidemia, *Cs* current smoking

Mean age was reported in years. Patients who were enrolled in this study had a mean age ranging from 60.0 to 70.0 years. Trials ITALIC [25], ISAR-SAFE [14] and ARCTIC [16] had a majority of males patients. Trial ISAR-SAFE [14] and study Thukkani2015 [23] involved a high number of patients with hypertension. According to the baseline features reported, no significant difference was observed among patients assigned to either a short or prolonged duration of DAPT use.

### Main analysis

Results of this analysis have been summarized in Table 4.

**Table 4:** Results of this analysis

Outcomes analyzed	OR with 95 % CI	*P* value	I^2^ (%)
Primary endpoints	1.03 [0.65–1.64]	0.90	0
Net clinical outcomes	0.96 [0.69–1.34]	0.81	0
Mortality	0.87 [0.76–1.00]	0.05	0
MI	0.85 [0.70–1.04]	0.12	0
TVR	0.85 [0.58–1.24]	0.39	0
TLR	0.90 [0.57–1.41]	0.63	0
Stroke	0.94 [0.65–1.36]	0.75	0
ST (definite or probable)	0.56 [0.27–1.17]	0.12	0
Definite ST	0.63 [0.08–4.79]	0.65	0
TIMI major bleeding	0.91 [0.60–1.37]	0.65	0
TIMI minor bleeding	1.08 [0.62–1.91]	0.78	0
BARC defined bleeding	1.92 [1.58–2.34]	0.00001	0
BARC type 2	1.98 [1.50–2.61]	0.00001	0
BARC type 3	1.78 [1.34–2.37]	0.0001	0
BARC type 5	1.40 [0.59–3.30]	0.44	0

*Abbreviations*: *OR* odds ratios, *CI* confidence intervals, *MI* myocardial infarction, *TVR* target vessel revascularization, *TLR* target lesion revascularization, *ST* stent thrombosis, *TIMI* thrombolysis in myocardial infarction, *BARC* bleeding academic research consortium

**Fig. 2:**
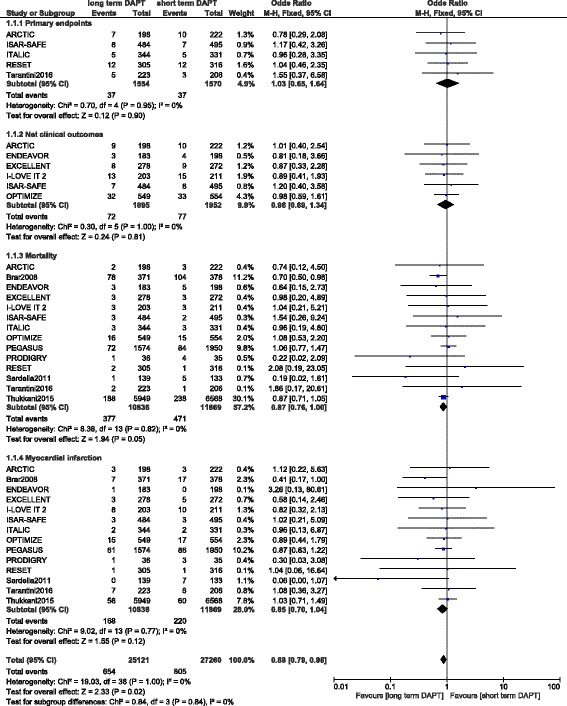
Adverse clinical outcomes associated with a short versus prolonged DAPT use in patients with DM (part 1)

This current analysis showed no significant differences in primary endpoints and net clinical outcomes in patients with DM whether with a short or prolonged treatment period with DAPT with OR: 1.03, 95 % CI: 0.65–1.64; *P* = 0.90 and OR: 0.96, 95 % CI: 0.69–1.34; *P* = 0.81 respectively. MI was also not significantly different with OR: 0.85, 95 % CI: 0.70–1.04; *P* = 0.12. However, even if mortality favored prolonged DAPT use, with OR: 0.87, 95 % CI: 0.76–1.00; *P* = 0.05, the result only approached statistical significance. These results have been illustrated in Fig. 2.

**Fig. 3:**
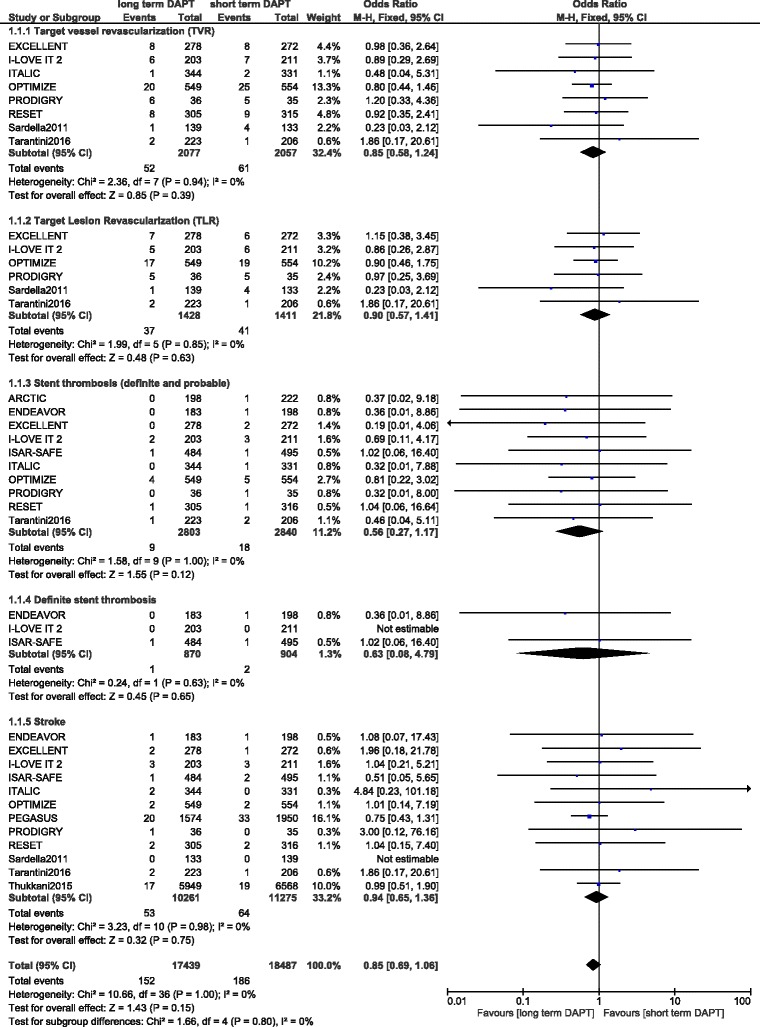
Adverse clinical outcomes associated with a short versus prolonged DAPT use in patients with DM (part 2)

TVR and TLR were also similarly manifested between these 2 groups with OR: 0.85, 95 % CI: 0.58–1.24; *P* = 0.39 and OR: 0.90, 95 % CI: 0.57–1.41; *P* = 0.63 respectively. Stroke was also not significantly different with a short term or prolonged DAPT use with these patients with DM, with OR: 0.94, 95 % CI: 0.65–1.36; *P* = 0.75. However, even if stent thrombosis favored a prolonged DAPT use with OR: 0.56, 95 % CI: 0.27–1.17; *P* = 0.12, this result was not statistically significant. These results have been illustrated in Fig. 3.

**Fig. 4:**
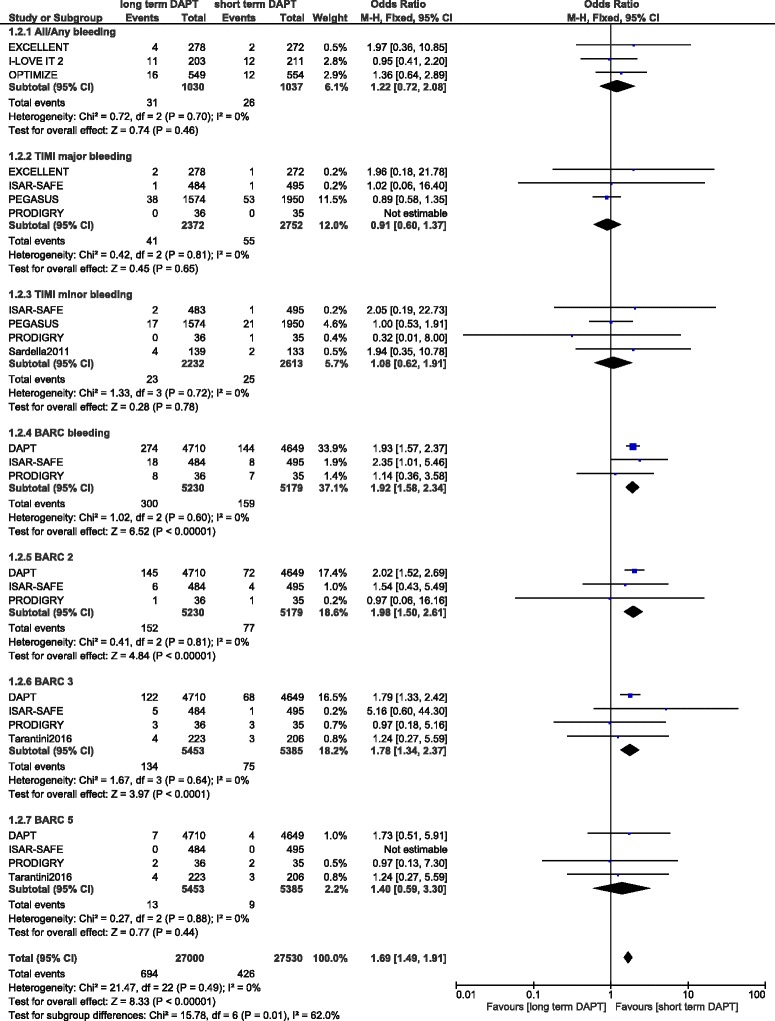
Bleeding events associated with a short versus prolonged DAPT use in patients with DM

Bleeding events were also analyzed in these patients with DM. Any bleeding was not significantly different between a short and a prolonged DAPT use with OR: 1.22, 95 % CI: 0.72–2.08; *P* = 0.46. TIMI defined major and minor bleeding were also not significantly different in these diabetic patients with OR: 0.91, 95 % CI: 0.60–1.37; *P* = 0.65 and OR: 1.08, 95 % CI: 0.62–1.91; *P* = 0.78 respectively. However, bleeding defined by the BARC classification was significantly higher with a prolonged DAPT use in these diabetic patients with OR: 1.92, 95 % CI: 1.58–2.34; *P* < 0.00001. When bleeding defined by BARC classification was further subdivided, a significantly higher BARC bleeding types 2 and 3 were observed with a prolonged DAPT use with OR: 1.98, 95 % CI: 1.50–2.61; *P* < 0.00001 and OR: 1.78, 95 % CI: 1.34–2.37; *P* < 0.0001 respectively. But even if BARC type 5 also favored a short term DAPT use, with OR: 1.40, 95 % CI: 0.59–3.30; *P* = 0.44, the result was not statistically significant. Results analyzing bleeding events have been illustrated in Fig. 4.

**Fig. 5:**
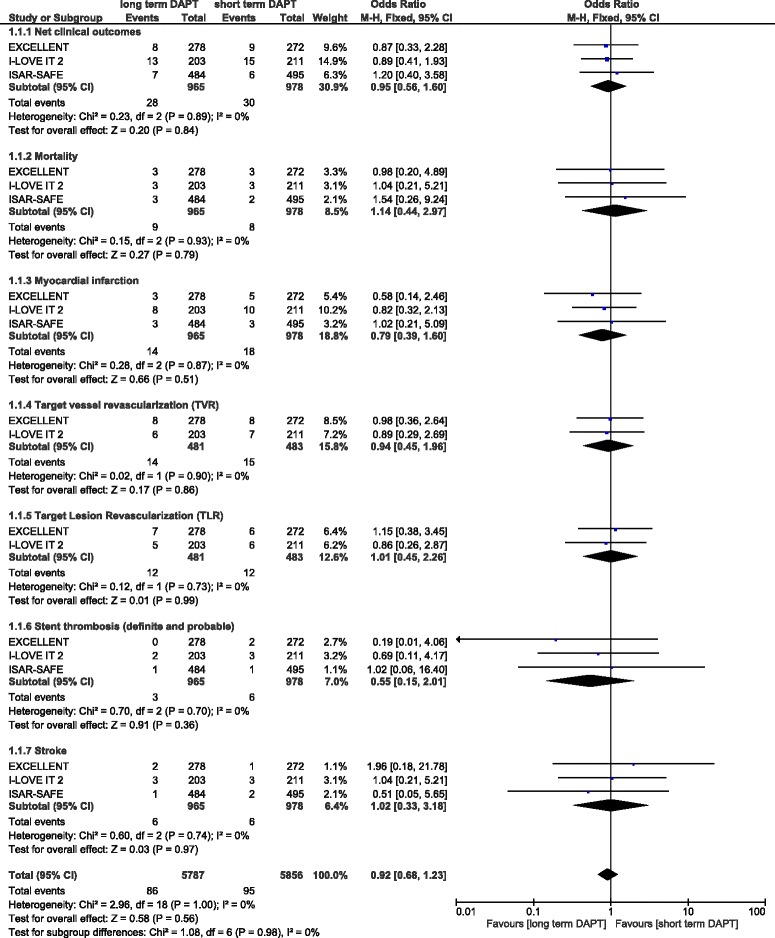
Adverse clinical outcomes associated specifically with 6 versus 12 months DAPT use in patients with DM

Because the duration period of DAPT was not similar in all the studies included, that is, a few studies had a short term DAPT duration period of 3 months, 6 months and 12 months respectively, and a long term DAPT duration period of 12 months, 18 months or 24 months respectively, which might have influenced the results of this analysis, another subgroup analysis was conducted only with a short term DAPT duration of 6 months versus a long term duration of 12 months. However, this analysis also showed no significant difference in net clinical outcomes, mortality, MI, TVR, TLR, stent thrombosis and stroke with OR: 0.95, 95 % CI: 0.56–1.60; *P* = 0.84, OR: 1.14, 95 % CI: 0.44–2.97; *P* = 0.79, OR: 0.79, 95 % CI: 0.39–1.60; *P* = 0.51, OR: 0.94, 95 % CI: 0.45–1.96; *P* = 0.86, OR: 1.01, 95 % CI: 0.45–2.26; *P* = 0.99, OR: 0.55, 95 % CI: 0.15–2.01; *P* = 0.36 and OR: 1.02, 95 % CI: 0.33–3.18; *P* = 0.97 respectively. These results comparing 6 months versus 12 months DAPT use have been illustrated in Fig. 5.

### Sensitivity analysis

**Fig. 6:**
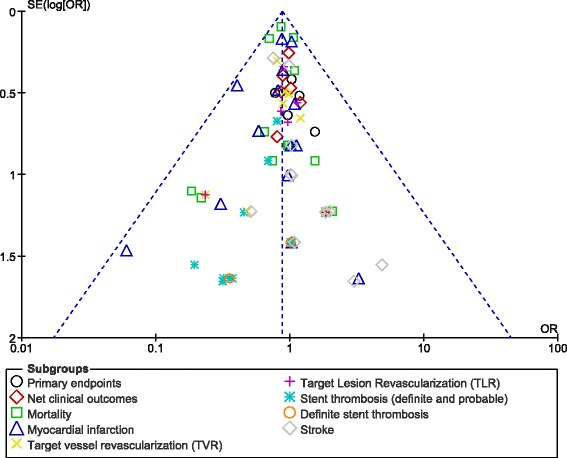
Funnel plots representing sensitivity analyses

**Fig. 7:**
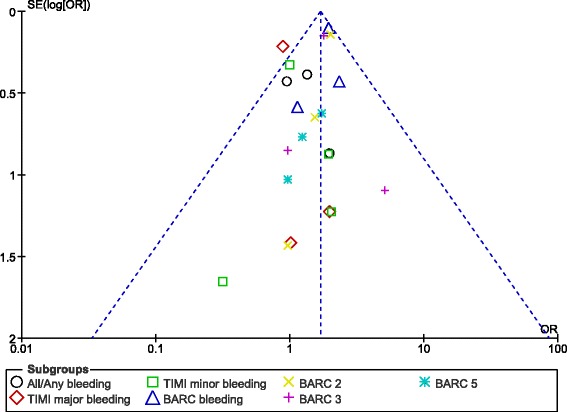
Funnel plots representing sensitivity analyses

For all of the above analyses, sensitivity analyses yielded consistent results. Based on a visual inspection of the funnel plots obtained, there has been very low evidence of publication bias for the included studies that assessed all clinical endpoints reported (including the adverse clinical outcomes and the bleeding events analyzed) in these patients with DM. The funnel plots have been illustrated in Figs. 6 and 7.

## Discussion

This study aimed to compare the adverse clinical outcomes associated with a short and prolonged DAPT use in patients with DM following PCI. Results of this study showed that a prolonged duration of DAPT use was not associated with any significant difference in adverse clinical outcomes when compared to a short term duration of DAPT use in these patients with DM. The result for mortality which favored a prolonged DAPT use reached near significance but was not statistically significant in this analysis whereas even if stent thrombosis favored a prolonged DAPT use, the result was also not statistically significant. In addition, TIMI defined major and minor bleeding were also not significantly different. However, bleeding defined according to BARC classification was significantly higher with the prolonged DAPT use.

In part similar to the results of this current analysis, the systematic review and meta-analysis comparing the duration of DAPT following DES implantation showed short term DAPT use to be associated with a significantly lower rate of bleeding, and higher rates of stent thrombosis [29]. Note that their study involved more than 30 % of patients with DM. However, their meta-analysis showed all-cause mortality to be insignificantly higher in the long term duration group, which was not the case in our study. In addition, this current study only showed a significantly increased bleeding rate according to the BARC classification, without any significant difference for stent thrombosis. Another meta-analysis published by Navarese et al. showed that compared to a DAPT duration period of 12 months, a short term DAPT use was associated with a significantly lower rate of bleeding events, without any apparent increase in ischemic complications and therefore the authors concluded that a short term DAPT could be considered in most patients following PCI [5].

Furthermore, the meta-analysis published by Yang et al. showed no difference in efficacy outcomes associated with a short or prolonged duration of DAPT use after intracoronary DES implantation [30]. However, a longer duration of DAPT (≥12 months) was associated with increased risk of bleeding complications. The study by Udell et al. also concluded that DAPT use beyond one year was associated with increased bleeding events, without any increase in cardiovascular mortality [4]. In addition, the PRODIGY trial which involved more than 20 % of patients with DM, showed a 24 months of clopidogrel use not to be associated with any increase in adverse clinical outcomes compared to the use of clopidogrel during a short term period of 6 months [31]. This trial compared device specific outcomes relative to different duration of DAPT in 3 different types of DES (everolimus eluting stents, paclitaxel eluting stents, zotarolimus eluting stents) and bare metal stents, suggesting that the optimal duration of DAPT could also possibly be stent specific.

Moreover, patients with DM showed comparable adverse clinical outcomes to that of patients without DM whether during a 6-months treatment with DAPT or a prolonged duration of DAPT following PCI with implanted second generation DES [15].

Nevertheless, the study by Valgimigli et al. showed that along with an increased risk of bleeding associated with a prolonged duration of DAPT use, an increased risk of stroke was also observed [32]. However, our results which involved patients with DM, did not show any significant difference in stroke rate between these two groups. The ITALIC trial also showed no significantly different bleeding or thrombotic events when 6 months DAPT use was compared to 24 months DAPT use after PCI [25]. However, the ITALIC trial involved patients implanted with newer generation DES and also involved patients with good response to aspirin.

The study by Siddiqqi et al. which consisted of more than 50 % of patients with DM, showed a prolonged duration of DAPT not to be associated with any increased risk of bleeding. However, their study involved patients with chronic kidney disease and the exact type of bleeding assessed was not specified [33]. The study by Thukkani et al. also showed results that favored the prolonged use of clopidogrel to be associated with a lower risk of death and MI only in patients with DM implanted with DES [23]. However, that study did not show any benefit of prolonged clopidogrel use in patients without DM or in patients implanted with bare metal stents. Moreover, the DAPT trial also showed a lower rate of mortality to be associated with a prolonged use of clopidogrel after PCI [20]. However, result of this analysis which involved only patients with DM, did not reach statistical significance in the subgroup analyzing mortality.

Even if the subgroup analysis of the OPTIMIZE trial that assessed how short-term DAPT did not show any significantly increase risk for clinical events at 1 year in patients with DM undergoing PCI with a specific 2nd generation DES [34], other studies have shown second generation DES to be associated with higher adverse outcomes in patients with DM compared to the general population [35]. In addition, studies showed increasing adverse clinical outcomes to be associated with insulin-treated DM compared to non-insulin treated DM irrespective of the duration of DAPT [36, 37].

Furthermore, the observational study conducted by Eisenstein et al. examining consecutive patients receiving DES at Duke Heart Center between the year 2000 and 2005, concluded that the extended use of clopidogrel in patients implanted with DES might be associated with a lower risk of death and MI [2]. However, the authors concluded that only larger trials will be able to confirm their results, but unfortunately, even if this current analysis involved a pooling of data from several randomized trials (but only including patients with DM), the result analyzing mortality only nearly reached statistical significance, but was not statistically significant.

Even if the result for stent thrombosis was not significant in patients with DM, other studies have shown a prolonged use of DAPT to be associated with a lower risk of stent thrombosis compared to a shorter duration of DAPT use after PCI. To prove this point, the TYCOON registry showed an extended use of DAPT (2 years) to be associated with a lower rate of stent thrombosis following PCI with DES [38]. However, in contrast, other studies showed clopidogrel use beyond one year not to reduce the risk of stent thrombosis or other adverse clinical outcomes after PCI [39].

Previous studies have already compared the clinical outcomes associated with duration of DAPT in the subset of patients treated for in-stent restenosis [23]. This current analysis showed compared the adverse clinical outcomes between a short and prolonged DAPT use in patients with DM. To be noted, the duration of DAPT use might vary in other subgroups of patients. As it is said, one size shoe approach for DAPT duration is unlikely to fit all the patients, further investigations including other subgroups of patients such as patients with chronic obstructive pulmonary disease who underwent PCI [40, 41], should be conducted.

### Novelty

This study is new in several ways. Even if many studies have compared the short and prolonged use of DAPT following PCI, this is among the first meta-analyses comparing the adverse clinical outcomes associated with the short and prolonged use of DAPT in patients with DM. Moreover, this analysis involved several newly published articles which were not included in other recently published meta-analyses representing another novelty.

### Limitations

Similar to other studies, this study also has limitations. First of all, due to the small population of patients with DM, this study might not provide robust results. Secondly, different studies reported different duration of DAPT use, as well as different follow up periods. Even if we have tried to compare only 6 months versus 12 months DAPT use in these patients with DM in order to solve this particular issue, we might have only partly succeeded showing that this point should still be considered as a limitation in this study. Moreover, data analyzing several bleeding subgroups were limited, which might have influenced the results.

## Conclusion

Following PCI, a prolonged DAPT use was associated with similar adverse clinical outcomes but with a significantly increased BARC defined bleeding compared to a short term DAPT use in these patients with DM. However, even if mortality and stent thrombosis favored a prolonged DAPT use, these outcomes only either reached statistical significance or were insignificant respectively, showing that a clear decision about recommending a prolonged duration of DAPT to patients with DM might not be possible at this moment, warranting further research in this particular subgroup.

## Abbreviations

ACS: Acute coronary syndrome
BARC: Bleeding academic research consortium
DAPT: Dual antiplatelet therapy
DES: Drug eluting stents
DM: Diabetes mellitus
PCI: Percutaneous coronary intervention
TIMI: Thrombolysis in myocardial infarction
